# The Association Between Hippocampal Volume and Level of Attention in Children and Adolescents

**DOI:** 10.3389/fnsys.2021.671735

**Published:** 2021-08-26

**Authors:** Tae-Hyeong Kim, Eunhye Choi, Hayeon Kim, Shin-Young Kim, Yeeun Kim, Bung-Nyun Kim, Subin Park, Kyu-In Jung, Bumhee Park, Min-Hyeon Park

**Affiliations:** ^1^Department of Psychiatry, College of Medicine, The Catholic University of Korea, Seoul, South Korea; ^2^Department of Psychiatry, Eunpyeong St. Mary's Hospital, College of Medicine, The Catholic University of Korea, Seoul, South Korea; ^3^Department of Psychiatry and Behavioral Science, Seoul National University College of Medicine, Seoul, South Korea; ^4^Department of Research Planning, National Center for Mental Health, Seoul, South Korea; ^5^Department of Biomedical Informatics, Ajou University School of Medicine, Suwon-si, South Korea; ^6^Office of Biostatistics, Ajou Research Institute for Innovative Medicine, Ajou University Medical Center, Suwon-si, South Korea

**Keywords:** hippocampal volume, continuous performance test, visual attention, auditory attention, children, adolescents

## Abstract

The hippocampus, which engages in the process of consolidating long-term memories and learning, shows active development during childhood and adolescence. The hippocampus also functionally influences attention. Based on the influence of hippocampal function on attention, it was expected that the volume of the hippocampus would be associated with the difference in attention during childhood and adolescence, in which the brain develops actively. Thus, this study examined the association between hippocampal volume and attention metrics measured by the continuous performance test (CPT) in 115 children and adolescents (mean age = 12.43 ± 3.0, 63 male and 52 female). In association studies with both auditory and visual attention, we found that the bilateral hippocampal volumes showed negative relationships with auditory omission errors. A smaller volume of the left hippocampus also led to a longer auditory response time. However, visual attention did not show any significant relationship with the hippocampal volume. These findings were consistent even after adjusting for the effects of the related covariates (e.g., age, insomnia, and depression). Taken together, this study suggested that the increase in hippocampal volume during childhood and adolescence was associated significantly with better auditory attention.

## Introduction

During childhood and adolescence, the brain develops substantially. The white matter volumes in brain regions of children and adolescents increase with their age (Giedd et al., 1999). The gray matter volumes (GMVs) in the frontal and parietal lobes peak during preadolescence and gradually decrease after adolescence (Giedd et al., 1999). While girls showed their maximized GMVs in the frontal and parietal lobes at the age of 11 years, the GMVs in these lobes were maximized at the age of 12.1 years in boys (Giedd et al., 1999). The volumes in the subcortical gray matter (i.e., hypothalamic gray matter, septal nuclei, some very anterior parts of the thalamus) and the mesiotemporal lobe (i.e., amygdala, hippocampus, uncus, and parahippocampal gyrus) also increase until early adulthood (i.e., age younger than 35 years) (Jernigan et al., 1991; Sowell and Jernigan, 1998). The hippocampus, which consists of four cornu ammon areas, the dentate gyrus and subiculum, shows active development during childhood and adolescence (De Bellis et al., 2000) in that the hippocampal volume starts to gradually decline from the early adulthood (Daugherty et al., 2016).

During childhood and adolescence, there is a sex difference in the white matter volume, where the white matter volume is larger in boys as compared with girls (Giedd et al., 1999). According to the previous studies, girls aged between 8 and 15 years show larger volumes in the bilateral hippocampus and the right striatum, but smaller volumes in the amygdala than boys (Giedd et al., 1997; Lenroot and Giedd, 2010).

The hippocampus, which plays an important role in the formation of long-term memories and learning (Lagali et al., 2010), engages in the encoding and retrieval stage of the memory consolidation process by modulating attention (Chun and Turk-Browne, 2007). To consolidate episodic memories, the hippocampus involves attention in the process and triggers the activation of the encoding stage and the retrieval stage. Two different hippocampal regions are activated during the encoding and retrieval stages. While the anterior portion of the hippocampus is activated in the encoding stage, the posterior portion of the hippocampus is activated in the retrieval stage (Lepage et al., 1998).

Although not always showing consistent results, the left hippocampus had a greater association with episodic memory compared with the right hippocampus (Shi et al., 2009), and the right hippocampus was more related to spatial memory. In addition, in a previous study of moving in mazes of various lengths (navigation phase) and estimating the direction of the starting point from the endpoint (pointing phase), the right hippocampus was activated during the navigation phase, and the left hippocampus was activated during the pointing phase. Activation of the right hippocampus was associated with better task performance, reflecting an effective spatial strategy, and activation of the left hippocampus reflected general mnemonic or verbal processing rather than performance (Persson et al., 2013).

Furthermore, attention prioritizes the information that is relevant to the goal of behaviors in the environment and modulates the cortex of the mesiotemporal region, where the input to the hippocampus is provided. Attention is also able to modulate the hippocampus by establishing a more detailed representation of the activity patterns in the various attentional states and stabilizing the distributed hippocampal representation of the task-relevant information (Aly and Turk-Browne, 2016a).

As it is suggested that attention engages in the memory consolidation process, the two different types of attention are involved in the encoding and retrieval stages depending upon the sensory input. One type of attention is perceptual attention, which refers to external attention. Perceptual attention selectively chooses and modulates sensory information (e.g., sight, sounds, and smell) based on task goals. The other type of attention is reflective attention, which is also known as internal attention. Reflective attention selects, sustains, and processes the information derived from the working and the long-term memories. In other words, perceptual attention engages in the encoding stage, and reflective attention engages in the retrieval stage during the memory consolidation process. Reflective attention is involved in the memory process through processing internal representations such as thoughts and memories without corresponding external stimuli or situations, and perceptual attention is involved in the memory process by processing external sensory stimuli (Chun and Johnson, 2011). Therefore, perceptual attention is closer to auditory and visual attention and is used to evaluate the level of attention.

Auditory attention and visual attention can be evaluated using auditory and visual stimuli. Similar to the “cocktail party effect,” where people can selectively focus on the voice of a specific communication partner among the various noisy conversations during a party (Taki et al., 2012), auditory attention helps to process the information through the auditory system by selectively paying attention to the relevant auditory stimuli and ignoring other stimuli (Kim, 2015). Although auditory attention is suggested to be influenced by the auditory cortex, auditory attention is also influenced by the right mid-thalamus and other brain regions (i.e., the frontal cortex, the bilateral precentral cortex, left postcentral cortex, and supplementary motor area) (Corbetta and Shulman, 2002). Visual attention refers to a mechanism that prioritizes the important information or the information relevant to survival by filtering the information in the visual system (Buckner et al., 2008). In addition to the parietal lobe, which is primarily involved in visual attention, other brain regions are found to engage in visual attention. While the superior colliculus of the midbrain is involved in eye movement and attention shift, the pulvinar nucleus of the thalamus, which is located near the lateral geniculate nucleus, plays an important role in controlling attention and filtering information (Buckner et al., 2008).

Although attention deficit hyperactivity disorder (ADHD) is a typical disorder associated with attention, there have been inconsistencies between the findings regarding the changes in hippocampal volume in children and adolescents who were diagnosed with ADHD (Plessen et al., 2006; Posner et al., 2014). The inconsistency between the findings suggested the plausible influence of comorbid disorder (e.g., depression) on hippocampal volume. That is, to examine the association between attention and changes in hippocampal volume in children and adolescents with ADHD, which is associated with inattention, more studies should be conducted, considering the covariates for clarification. Furthermore, unlike the study that demonstrated the association of attention with the function of the hippocampus by finding that adults, who were better at recalling working memory, showed higher activation in the hippocampus during the encoding stage (Aly and Turk-Browne, 2016a), the association of hippocampal volume with attention during childhood and adolescence was less examined.

Thus, this study aimed to examine the correlation between hippocampal volume and attention in children and adolescents aged between 6 and 18 years. As perceptual attention was found to select and modulate sensory information compared to reflective attention through the continuous performance test (CPT), this study evaluated visual attention and auditory attention, which are closer to perceptual attention. Factors that plausibly influenced hippocampal volume (e.g., sleep problems and depression) were also considered.

## Materials and Methods

### Participants

We recruited children and adolescents ranging from 6 to 18 years of age *via* a flier posted in schools and libraries in Seoul and Gyeonggi-do Province. Initially, a total of 150 participants were recruited (age = 11.9 ± 3.1, male = 79, female = 71) for the study. All the participants who were engaged in this study met the following criteria: (i) they were capable of understanding and following the instructions and descriptions fully in the present study; (ii) they had no possibility of pregnancy for female before the study; (iii) they were taking no medications that could significantly influence their waking and sleep conditions; and (iv) they had no predicted problems in neuroimaging and psychological tests. Participants with any clear neuropsychiatric history of psychiatric disorders (schizophrenia, bipolar disorder, or pediatric psychosis), developmental disorders (autism or intellectual disabilities), learning disabilities, language impairments, uncorrected sensory impairments, neurological disorders (convulsive disorder), or acquired brain injury (cerebral palsy) were excluded from the analyses (Sung et al., 2020).

Of the 150 participants, 35 were excluded from the study because of failure of the MRI scan or failure to complete the survey or the CPT test. Therefore, data from 115 participants were analyzed for this study (age = 12.4 ± 3.0 years, male = 63, female = 52).

This study was approved by the Institutional Review Board (IRB) for Human Subjects at the Seoul National University Hospital (No. C-1412-081-633) and was conducted in accordance with the 1964 Declaration of Helsinki. All the participants and their parents or legal guardians completed the written informed consent.

### Materials

#### IQ

##### Korean Educational Developmental Institute-Wechsler Intelligence Scale for Children

The KEDI-WISC is a modified version of the revised Wechsler Intelligence Scale for Children [WISC-R; (Wechsler, 1974)] used in Korea. This test was developed through a standardization process in Korea to measure the intelligence of children from 5 to 15 years old. The KEDI-WISC comprises 10 subtests (five verbal scales consisting of vocabulary, information, comprehension, arithmetic, and similarities subtests; five performance scales consisting of block design, picture completion, picture arrangement, digit symbol, and object assembly subtests) and two additional subtests (verbal scale, digit span; performance scale, maze). In this study, only the digit span test of the additional tests was used (Institute, 1987).

##### Korean–Wechsler Adult Intelligence Scale

The K–WAIS is a Korean version of the revised Wechsler Adult Intelligence Scale (WAIS-R) (Wechsler, 1981). It is used for assessing the intelligence of adolescents and adults between the ages of 16 and 64. This test comprises 11 tests (verbal and performance tests). Approximately 6-Verbal subtests include digit span, information, vocabulary, arithmetic, comprehension, and similarities, while 5-performance subtests include block design, picture completion, picture arrangement, digit symbol, and object assembly (Association, 1992).

##### Continuous Performance Test

The Advanced test of attention (ATA) is a CPT consisting of visual and auditory attention tests. The ATA was developed to measure attention and response inhibition in Korean children over 5 years of age. The time of stimulus presentation was 0.1 s, and the interval of each stimulus was 2 s for 15 min (Cho et al., 2000). The target rate of stimulus presentation was 22% in the beginning, 50% in the middle, and 78% in the second half (Cho et al., 2000). The ATA measured age-adjusted *T* scores based on four variables: commission errors (CE), omission errors (OE), mean reaction time (RT), and response time variability (RTv). A commission error is a measure of impulsivity, inhibitory control, and self-regulation and refers to when the patient responded to a non-target stimulus. An omission error is a measure of sustained attention and refers to when the patient did not respond to a target stimulus. The mean reaction time indicates the response preparation components of the executive function. The response time variability assesses the inconsistency in the responses of the patients.

According to the ATA guidelines, a *T* score above 65 on visual-auditory variables means some probability of ADHD. Therefore, a *T* score of 65 as our cutoff score was used to distinguish between normal and abnormal performances in this study.

##### Insomnia Severity Index

The ISI was developed by Morin and colleagues (Morin, 1993) and is a 7-item scale that assesses the severity of insomnia symptoms, satisfaction with sleep, interference with daytime functioning, awareness of impairment, and concern about sleep problems (Bastien et al., 2001). The respondents described the severity of their insomnia over the past 2 weeks on a 5-point Likert scale ranging from 0 to 4.

##### The Children's Depression Inventory

The CDI is a self-report scale that has been modified from Beck's depression inventory for children, and we utilized the Korean version (Cho and Lee, 1990). The CDI assesses the level of depression in 7- to 17-year-old children and adolescents. This scale, composed of 27 items, is rated from 0 to 2 points according to severity. The total score ranges from 0 to 54 points, and a higher score indicates more severe depression (Kovacs, 1985).

##### MRI Data Acquisition

MRIs were collected using a 3.0 Tesla MRI scanner (Siemens, Magnetom Tim-Trio). We obtained high-resolution T1-weighted images using a magnetization-prepared rapid acquisition gradient echo (MPRAGE) pulse sequence [repetition time (TR) = 1900 ms; echo time (TE) = 3.13 ms; flip angle = 9°; matrix size = 256 x 256; field of view (FOV) = 230 x 230 mm^2^; thickness = 0.9 mm]. We used foam pads to minimize head motion-related artifacts during scanning.

##### Regional Voxel-Based Morphometry Analysis

To measure the bilateral hippocampal volumes, we performed a VBM analysis using the SPM12 VBM-DARTEL procedure (SPM12, http://www.fil.ion.ucl.ac.uk/spm/, Wellcome Trust Centre for Neuroimaging, London, UK) (Ashburner, 2007). This can improve the registration and make the segmentation between different tissues more obvious compared with the previous optimized VBM (Ashburner, 2007; Takao et al., 2010).

We inspected T1-weighted images carefully for motion and/or other artifacts. No abnormalities due to artifacts were found. The procedure included the following steps for preprocessing T1-weighted images. The methods have been described in detail in previous studies. (Jung et al., 2019; Sung et al., 2020): (1) the images were manually reoriented to the anterior commissure, (2) the gray matter was segmented using a standard tissue probability map provided by SPM, (3) using the DARTEL procedure, all the images were created as a study-specific template, (4) the images were spatially normalized using the DARTEL template, and the brain size of individuals was adjusted during spatial normalization, and (5) the gray matter was smoothed with a Gaussian kernel of 8 mm full-width at half maximum. After preprocessing, the regional GMV (rGMV) was extracted by averaging the values in the left and right hippocampi, which were defined using the automated anatomical labeling (AAL) atlas.

In this study, we adopted a standard adult SPM template instead of an age-specific template (Tzourio-Mazoyer et al., 2002). Previous neuroimaging studies on developing brains have suggested that neuroanatomical differences between adults and even children do not influence the results even after using the adult template (Muzik et al., 2000; Burgund et al., 2002; Kang et al., 2003; Poldrack, 2010; Richards et al., 2016). Using the standard adult SPM template enables us to compare or combine the results from previous literature with adults or across the different age groups (Weiss and Booth, 2017; Ross et al., 2019; Verdejo-Román et al., 2019).

### Statistical Analysis

We carried out partial correlation analyses to investigate associations of hippocampal rGMVs with visual and auditory attention-related clinical variables [covariates: age, sex, total intracranial volume (TIV), and IQ].

A threshold false discovery rate (FDR) = 0.2 was determined to be significant for addressing multiple comparison issues (i.e., FDR = 0.2 or less for significance) (Genovese et al., 2002). Only among brain areas showing significance does FDR thresholding optimize the expected proportion of false positives (Genovese et al., 2002). FDR levels in the range of 0.1–0.2 are practically and originally known to be acceptable, as they have been applied in several neuroimaging studies (Genovese et al., 2002; Molteni et al., 2017; Gordon et al., 2018; Yu et al., 2018; Jung et al., 2019). All statistical analyses were performed using SPSS 20.0 for Windows (SPSS Inc., Chicago, IL, USA) and MATLAB-based custom software (MathWorks, Sherborn, MA, USA).

## Results

### Demographic and Clinical Characteristics of the Participants

The study evaluated 115 participants (age = 12.4 ± 3.0, male = 63, female = 52). All the participants were assessed for IQ, CDI, ISI, and CPT, and a brain MRI was performed. Then, we measured the bilateral hippocampal volume through VBM analysis with the obtained brain images. Participants were classified by sex and age (based on 12 years), and clinical characteristics were analyzed for this study (Table 1).

**Table 1:** Demographic and clinical characteristics of the participants.

**Characteristics**	**Mean** **±** **SD**			***P-*** **values**	***P-*** **values (Adjusted by age)**	**Mean** **±** **SD**		***P-*** **values**	***P-*** **values** **(Adjusted by sex)**
	**Total** **(*N* = 115)**	**Male** **(*n* = 63)**	**Female** **(*n* = 52)**			**Age <12** **(*n* = 44)**	**Age ≥ 12** **(***n*** = 71)**		
Age	12.4 ± 3.0	12.7 ± 3.2	12.0 ± 2.7	0.194	-	9.1 ± 1.5	14.5 ± 1.4	-	-
IQ	102.3 ± 16.5	98.9 ± 16.2	106.5 ± 16.0	0.012	0.019	103.9 ± 18.7	101.2 ± 14.9	0.395	0.464
ISI	7.4 ± 4.6	7.6 ± 4.3	7.2 ± 4.9	0.589	0.554	7.8 ± 4.2	7.2 ± 4.8	0.506	0.488
CDI	14.6 ± 8.0	15.2 ± 8.2	13.9 ± 7.9	0.404	0.943	9.7 ± 6.1	17.7 ± 7.6	<0.0001	<0.0001
ATAa	127 ± 23.3	126.6 ± 23.1	127.5 ± 23.7	0.841	0.894	129 ± 21.5	125.7 ± 24.4	0.455	0.462
ATAa_OE	71.7 ± 22.2	71.5 ± 22.3	72.1 ± 22.3	0.886	0.927	73.0 ± 20.4	70.9 ± 23.4	0.608	0.614
ATAa_CE	72.2 ± 21.1	71.5 ± 21.0	73.0 ± 21.3	0.698	0.921	77.5 ± 19.2	68.7 ± 21.6	0.026	0.023
ATAa_RT	49.9 ± 15.5	49.5 ± 16.9	50.4 ± 13.8	0.741	0.216	40.3 ± 15.6	56.1 ± 11.9	<0.0001	<0.0001
ATAa_RTv	49.7 ± 10.0	49.2 ± 9.7	50.4 ± 10.4	0.523	0.616	50.9 ± 9.9	48.9 ± 10.1	0.312	0.329
ATAv	121 ± 22.5	123.8 ± 24.2	117.6 ± 19.9	0.132	0.045	128.1 ± 25.8	116.4 ± 18.9	0.006	0.004
ATAv_OE	59.3 ± 19.6	62.1 ± 21.6	55.7 ± 16.2	0.071	0.008	70.3 ± 21.8	52.2 ± 14.1	<0.0001	<0.0001
ATAv_CE	64.7 ± 19.3	65.7 ± 19.9	63.6 ± 18.7	0.564	0.306	69.5 ± 21.4	61.6 ± 17.3	0.03	0.027
ATAv_RT	60.4 ± 14.4	61.9 ± 13.9	58.5 ± 14.9	0.211	0.335	56.8 ± 12.3	62.7 ± 15.2	0.032	0.037
ATAv_RTv	56.6 ± 19.9	58.4 ± 21.4	54.4 ± 17.8	0.275	0.116	62.7 ± 19.4	52.7 ± 19.3	0.007	0.006

*ATAa, advanced test of attention – auditory; ATAv, advanced test of attention – visual; OE, omission errors; CE, commission errors; RT, mean response time; RTv, response time variability*.

When participants were divided by sex (male = 63, female = 52), the female participants showed better performance on the IQ test (*P* < 0.012). After adjusting for age, the differences in clinical characteristics between male and female were not significant except for IQ (*P* < 0.019), visual ATA (ATAv) (*P* < 0.045), and ATAv_omission errors (OE) (*P* < 0.008).

When the analysis was conducted by subgrouping children and adolescents [based on 12 years, age < 12 (*n* = 44), age ≥ 12 (*n* = 71)], the CDI score showed a high score in the age group of 12 years and older (*P* < 0.0001). In the auditory attention evaluation, participants above 12 years had lower results in ATAa_comission errors (CE) (*P* = 0.026), but had slower results in ATAa_RT (mean of response time) (*P* < 0.0001) than the other group. In the visual attention evaluation, participants above 12 years showed lower ATAv (*P* = 0.006), ATAv_OE (*P* < 0.0001), ATAv_CE (*P* = 0.03) and ATAv_Response Time variability (ATAv_RTv) scores (*P* = 0.007). On the contrary, the participants under 12 years had faster results in ATAv_RT (*P* = 0.032). After adjusting for sex, all the results were significant.

### Correlation Analysis Between Regional GMV and CPT Score

**Figure 1:**
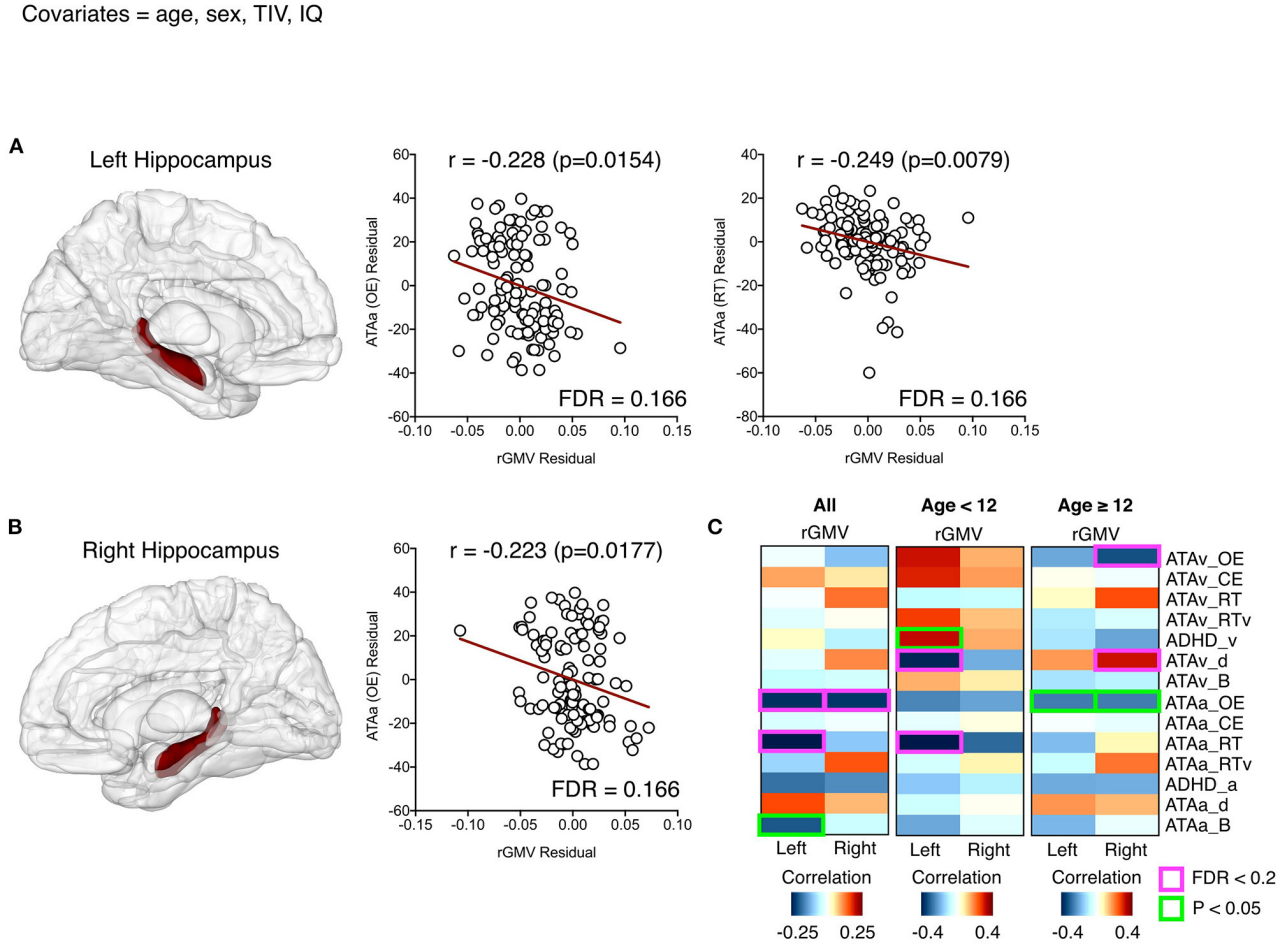
Significant relationships between the left **(A)** and right **(B)** hippocampal volumes and attention-related variables (partial correlation; covariates = age, sex, total intracranial volume, and IQ). The first columns in **(A)** and **(B)** each represent the hippocampal regions defined by the automated anatomical labeling atlas. Residuals at scatter plots were calculated after partial correlation analyses, and red lines represent predicted linear regression lines. All the correlations between two regional volumes and all attention-related variables are described in **(C)**, where red boxes and text indicate a significant relationship with a false discovery rate < 0.2.

The auditory ATA_OE score showed negative correlations with both the left and right hippocampal volumes, while the visual ATA_OE score did not show any correlation (Figures 1A,B). The auditory ATA_RT score was also negatively correlated with the left hippocampal volume (Figure 1B). These results indicate that the larger the hippocampal volumes of both, the better the auditory ATA_OE performance and the faster the mean response time.

We described the two regional volumes with a significant correlation (FDR < 0.2) between variables related to attention and regional GMV.

Analysis of children and adolescents based on 12-year-old showed that the left hippocampal volume of participants under 12 years was negatively correlated with auditory ATA_RT score and visual ATA_detectability (d). This means that the left hippocampal volume is large and the mean response time is faster, but the detectability for visual ATA is low, which means that it is difficult to distinguish the target stimulus from the non-target stimulus.

For the participants aged 12 years or older, the right hippocampal volume was negatively correlated with visual ATA_OE and showed positive correlations with visual ATA_d(detectability). The volume of the right hippocampus affects visual ATA_OE performance. The larger the right hippocampal volume is, the better the stimuli can be distinguished in the visual ATA (Figure 1C).

The mean hippocampal volume between boys and girls was adjusted for age, TIV, and IQ. This adjustment did not change the results (left HP: *P* = 0.6210, right HP: *P* = 0.4485). We represented all partial correlation values and the significant values, including both uncorrected and FDR-adjusted *p*-values, in Supplementary Table 1.

Since hippocampal volumes are affected by sleep or depression, we examined additional effects of sleep (ISI) and depression-related scales (CDIs) by including those covariates in the partial correlation analyses. This adjustment did not significantly change the results shown in Figure 1.

An additional adjustment for age, which involved separate analyses of children (12-year-old or younger) and adolescents (13 years or older), also did not change the results.

## Discussion

This study aimed to examine the association of hippocampal volume with visual and auditory attention in children and adolescents. These findings suggest that larger bilateral hippocampal volumes performed better by showing fewer omission errors in response to auditory stimuli. In addition, the larger volume in the left hippocampus responded more quickly to auditory stimuli presented in the ATA after factors that seemed to influence the volume of the hippocampus, such as sleep, depressive symptoms, age, IQ, sex, and TIV (Videbech and Ravnkilde, 2004; Taki et al., 2012), were adjusted in the analysis.

In this study, there was no significant sex difference in hippocampal volume after adjustment for covariates (e.g., age, TIV, and IQ). Although female were found to show a larger volume in the hippocampus than male between the ages of 8 and 15 years (Lenroot and Giedd, 2010), there was no sex difference in the hippocampal volume in the participants aged between 4 and 18 years after the adjustment of the total cerebral volume (Giedd et al., 1997), which was consistent with the results of this study.

While the anterior hippocampus was found to be more activated in the encoding stage of the memory consolidation process, the posterior hippocampus was found to be more engaged in the retrieval stage (Kim, 2015). Furthermore, brain regions that were activated during the encoding stage were associated with the dorsal attention network that activates during externally oriented activities (e.g., sense and visual direction, the detection of the goal, and the selection of sensory response) (Corbetta and Shulman, 2002). Activated brain regions during the retrieval stage were associated with the default mode network, which showed increased activation in internal mental activity, such as recall, the imagination of the future, mentalization, and visual representation (Buckner et al., 2008). In addition, the encoding effect refers to the process where the left-lateralized region is involved in the cognitive control network to process the meanings of relevant information for effective memory consolidation, and the subsequent memory (SM) effect was found to be larger when encoding verbal materials than pictorial materials. Activation in the left lateral region was also suggested to influence the dorsal attention network by making the cognitive control network focus on external attention (Kim, 2015). That is, various perceptual stimuli might increase the activation in the anterior portion of the hippocampus by being involved in the activation of the dorsal attention network. The left region might exert a greater influence on the extent of attention in that the left lateral region is more activated by auditory stimuli than by visual stimuli during the encoding stage and cooperates with the dorsal attention network. Taken together, these studies supported the findings of this study that the left hippocampus was more involved in auditory attention.

Furthermore, while the anterior part of the hippocampus was found to show more activation in the auditory oddball task, the posterior part of the hippocampus was activated in the visual oddball task (Crottaz-Herbette et al., 2005). The activated portion of the hippocampus in the auditory oddball task overlaps with the part of the hippocampus that perceptual attention activates in selecting and processing location and sensory information (Chun and Johnson, 2011). The results also supported the results of the current study that the hippocampus is more associated with auditory attention than with visual attention.

In a study that used a verbal memory task to examine the association of hippocampal volume with auditory attention in adults who survived after the diagnosis of brain tumors in the posterior fossa, pituitary lobe, frontal lobe, and temporal lobe during childhood, adult patients with lower volumes in the bilateral hippocampus showed significantly poorer auditory attention than healthy controls (Jayakar et al., 2015). The more sensitive response of the left hippocampus to linguistic stimuli (Jayakar et al., 2015) supported our results that participants with larger volumes in the left hippocampus showed shorter response times to auditory stimuli.

Although it was found that the activation of attention became larger in the parahippocampal cortex (PHc), perirhinal cortex (PRc) and entorhinal cortex (ERc) in the presentation of visual stimuli, whether the hippocampus is involved in the activation of attention in response to visual stimuli is not known (Dudukovic et al., 2011; Aly and Turk-Browne, 2016b). Visual attention was found to be closely associated with activation in parietal areas, including the superior parietal lobule (SPL) and intraparietal sulcus (IPS) (Wojciulik and Kanwisher, 1999). Consistent with these findings, hippocampal volume was not significantly correlated with visual attention in this study. Taken together, these results suggested that visual attention seemed to be associated not with the hippocampus but instead with the parietal area.

In this study, it was found that the volume of the hippocampus was positively associated with attention. However, when the change in hippocampal volume depending on subtypes of ADHD in children and adolescents was examined, there was no significant difference in hippocampal volume between children and adolescents with inattentive ADHD and healthy controls (Al-Amin et al., 2018). The inconsistency between this study and the study including those with ADHD seemed to result from the influence of medication for ADHD. As children and adolescents with ADHD received medication to improve ADHD, it was plausible that there was no significant difference in hippocampal volume between the children and adolescents with ADHD and the healthy controls.

There were two limitations to this study. One limitation was the design of the study. As the design of this study was cross-sectional, it was not clear whether the structural change in hippocampal volume was accompanied by changes in auditory attention. That is, this study provided a limited explanation for the correlation between hippocampal volume and auditory attention. The other limitation was the issue of the representative sample. As the participants were recruited in Seoul and Gyeonggi-do Province, selection bias might have occurred during recruitment.

Despite these two limitations, this study is clinically useful. To our knowledge, this is the first study to examine the association between attention and hippocampal volume in the typically developing brains of children and adolescents using CPT. As the association of the hippocampus with attention has been underestimated, this study suggested that hippocampal volume is plausibly associated with attention during childhood and adolescence, in which the brain actively develops.

To clarify the correlation of hippocampal volume with attention, further prospective studies should be conducted using longitudinal observations. Furthermore, conducting additional studies to examine the association between the volumes of the hippocampal subfields that are divided based on the anatomical and functional differences (Duvernoy, 2005), and the extent of attention would help to clarify how different subfields of the hippocampus are correlated with attention.

## Data Availability Statement

The raw data supporting the conclusions of this article will be made available by the authors, without undue reservation.

## Ethics Statement

The studies involving human participants were reviewed and approved by the Institutional Review Board (IRB) for Human Subjects at Seoul National University Hospital (No. C-1412-081-633). Written informed consent to participate in this study was provided by the participants' legal guardian/next of kin. Written informed consent was obtained from the individual(s), and minor(s)' legal guardian/next of kin, for the publication of any potentially identifiable images or data included in this article.

## Author Contributions

M-HP designed the study and wrote the protocol. EC, HK, S-YK, YK, B-NK and SP managed the literature searches and analyses. BP and S-YK undertook the statistical analysis, and T-HK, HK, K-IJ, M-HP, and BP wrote the first draft of the manuscript. All the authors contributed to and approved the final manuscript.

## Conflict of Interest

The authors declare that the research was conducted in the absence of any commercial or financial relationships that could be construed as a potential conflict of interest.

## Publisher's Note

All claims expressed in this article are solely those of the authors and do not necessarily represent those of their affiliated organizations, or those of the publisher, the editors and the reviewers. Any product that may be evaluated in this article, or claim that may be made by its manufacturer, is not guaranteed or endorsed by the publisher.

## References

[B1] Al-Amin M. Zinchenko A. Geyer T. (2018). Hippocampal subfield volume changes in subtypes of attention deficit hyperactivity disorder. Brain Res. 1685, 1–8. 10.1016/j.brainres.2018.02.007 29427578

[B2] Aly M. Turk-Browne N. B. (2016a). Attention promotes episodic encoding by stabilizing hippocampal representations. Proc. Natl. Acad. Sci. U. S. A. 113, E420–429. 10.1073/pnas.1518931113 26755611 PMC4743819

[B3] Aly M. Turk-Browne N. B. (2016b). Attention stabilizes representations in the human hippocampus. Cereb. Cortex 26, 783–796. 10.1093/cercor/bhv041 25766839 PMC4712804

[B4] Ashburner J. (2007). A fast diffeomorphic image registration algorithm. J. Neuroimage 38, 95–113. 10.1016/j.neuroimage.2007.07.007 17761438

[B5] Association K. C. P. (1992). K-WAIS Guidance. Seoul: Korea Guidance.

[B6] Bastien C. H. Vallières A. Morin C. M. (2001). Validation of the insomnia severity index as an outcome measure for insomnia research. Sleep Med. 2, 297–307. 10.1016/S1389-9457(00)00065-4 11438246

[B7] Buckner R. L. Andrews-Hanna J. R. Schacter D. L. (2008). The brain's default network: anatomy, function, and relevance to disease. Ann. N. Y. Acad. Sci. 1124, 1–8. 10.1196/annals.1440.011 18400922

[B8] Burgund E. D. Kang H. C. Kelly J. E. Buckner R. L. Snyder A. Z. Petersen S. E.. (2002). The feasibility of a common stereotactic space for children and adults in fMRI studies of development. Neuroimage 17, 184–200. 10.1006/nimg.2002.1174 12482076

[B9] Cho S.-Z. Chun S.-Y. Hong K.-E. Shin M.-S. (2000). A study of the development and standardization of ADHD diagnostic system. J. Korean Acad. Child Adolesc. Psychiatry 11, 91–99.

[B10] Cho S. C. Lee Y. S. (1990). Development of the Korean form of the Kovacs' children's depression inventory. J. Korean Neuropsychiatr. Assoc. 29, 943–956.

[B11] Chun M. M. Johnson M. K. (2011). Memory: enduring traces of perceptual and reflective attention. Neuron 72, 520–535. 10.1016/j.neuron.2011.10.026 22099456 PMC3248396

[B12] Chun M. M. Turk-Browne N. B. (2007). Interactions between attention and memory. Curr. Opinion Neurobiol. 17, 177–184. 10.1016/j.conb.2007.03.005 17379501

[B13] Corbetta M. Shulman G. L. (2002). Control of goal-directed and stimulus-driven attention in the brain. Nat. Rev. Neurosci. 3, 201–215. 10.1038/nrn755 11994752

[B14] Crottaz-Herbette S. Lau K. M. Glover G. H. Menon V. (2005). Hippocampal involvement in detection of deviant auditory and visual stimuli. Hippocampus 15, 132–139. 10.1002/hipo.20039 15390157

[B15] Daugherty A. M. Bender A. R. Raz N. Ofen N. (2016). Age differences in hippocampal subfield volumes from childhood to late adulthood. Hippocampus 26, 220–228. 10.1002/hipo.22517 26286891 PMC4718822

[B16] De Bellis M. D. Clark D. B. Beers S. R. Soloff P. H. Boring A. M. Hall J.. (2000). Hippocampal volume in adolescent-onset alcohol use disorders. Am. J. Psychiatry 157, 737–744. 10.1176/appi.ajp.157.5.737 10784466

[B17] Dudukovic N. M. Preston A. R. Archie J. J. Glover G. H. Wagner A. D. (2011). High-resolution fMRI reveals match enhancement and attentional modulation in the human medial temporal lobe. J. Cogn. Neurosci. 23, 670–682. 10.1162/jocn.2010.21509 20433244 PMC5746189

[B18] Duvernoy H. M. (2005). The human hippocampus: functional anatomy, vascularization and serial sections with MRI. Springer Sci. Bus. Media. 10.1007/b138576

[B19] Genovese C. R. Lazar N. A. Nichols T. (2002). Thresholding of statistical maps in functional neuroimaging using the false discovery rate. Neuroimage 15, 870–878. 10.1006/nimg.2001.1037 11906227

[B20] Giedd J. N. Blumenthal J. Jeffries N. O. Castellanos F. X. Liu H. Zijdenbos A.. (1999). Brain development during childhood and adolescence: a longitudinal MRI study. Nat. Neurosci. 2, 861–863. 10.1038/13158 10491603

[B21] Giedd J. N. Castellanos F. X. Rajapakse J. C. Vaituzis A. C. Rapoport J. L. (1997). Sexual dimorphism of the developing human brain. Prog. Neuro Psychopharmacol. Biol. Psychiatry 21, 1185–1201. 10.1016/S0278-5846(97)00158-9 9460086

[B22] Gordon B. A. McCullough A. Mishra S. Blazey T. M. Su Y. Christensen J.. (2018). Cross-sectional and longitudinal atrophy is preferentially associated with tau rather than amyloid β positron emission tomography pathology. Alzheimer's Dement. 10, 245–252. 10.1016/j.dadm.2018.02.003 29780869 PMC5956934

[B23] Institute K. E. D. (1987). KEDI-WISC test outline by Korea Educational Development Institute. Seoul: Special Education Publishing Company.

[B24] Jayakar R. King T. Z. Morris R. Na S. (2015). Hippocampal volume and auditory attention on a verbal memory task with adult survivors of pediatric brain tumor. Neuropsychology 29, 303–319. 10.1037/neu0000183 25643218

[B25] Jernigan T. L. Trauner D. A. Hesselink J. R. Tallal P. A. (1991). Maturation of human cerebrum observed in vivo during adolescence. Brain 114, 2037–2049. 10.1093/brain/114.5.2037 1933232

[B26] Jung K.-I. Park M.-H. Park B. Kim S.-Y. Kim Y. O. Kim B.-N.. (2019). Cerebellar gray matter volume, executive function, and insomnia: gender differences in adolescents. Sci. Rep. 9:855. 10.1038/s41598-018-37154-w 30696877 PMC6351545

[B27] Kang H. C. Burgund E. D. Lugar H. M. Petersen S. E. Schlaggar B. L. (2003). Comparison of functional activation foci in children and adults using a common stereotactic space. Neuroimage 19, 16–28. 10.1016/S1053-8119(03)00038-7 12781724

[B28] Kim H. (2015). Encoding and retrieval along the long axis of the hippocampus and their relationships with dorsal attention and default mode networks: The HERNET model. Hippocampus 25, 500–510. 10.1002/hipo.22387 25367784

[B29] Kovacs M. (1985). The Children's Depression, Inventory (CDI). Psychopharmacol. Bull. 21, 995–998. 4089116

[B30] Lagali P. S. Corcoran C. P. Picketts D. J. (2010). Hippocampus development and function: role of epigenetic factors and implications for cognitive disease. Clin. Genet. 78, 321–333. 10.1111/j.1399-0004.2010.01503.x 20681996

[B31] Lenroot R. K. Giedd J. N. (2010). Sex differences in the adolescent brain. Brain Cogn. 72, 46–55. 10.1016/j.bandc.2009.10.008 19913969 PMC2818549

[B32] Lepage M. Habib R. Tulving E. (1998). Hippocampal PET activations of memory encoding and retrieval: the HIPER model. Hippocampus 8, 313–322. 10.1002/(SICI)1098-1063(1998)8:4<313::AID-HIPO1>3.0.CO;2-I 9744418

[B33] Molteni E. Rocca M. A. Strazzer S. Pagani E. Colombo K. Arrigoni F.. (2017). A diffusion tensor magnetic resonance imaging study of paediatric patients with severe non-traumatic brain injury. Dev. Med. Child Neurol. 59, 199–206. 10.1111/dmcn.13332 27910995

[B34] Morin C. M. (1993). Insomnia: Psychological assessment and management. Guilford Press.

[B35] Muzik O. Chugani D. C. Juhász C. Shen C. Chugani H. T. (2000). Statistical parametric mapping: assessment of application in children. Neuroimage 12, 538–549. 10.1006/nimg.2000.0651 11034861

[B36] Persson J. Herlitz A. Engman J. Morell A. Sjölie D. Wikström J.. (2013). Remembering our origin: gender differences in spatial memory are reflected in gender differences in hippocampal lateralization. Behav. Brain Res. 256, 219–228. 10.1016/j.bbr.2013.07.050 23938766

[B37] Plessen K. J. Bansal R. Zhu H. Whiteman R. Amat J. Quackenbush G. A.. (2006). Hippocampus and Amygdala Morphology in Attention-Deficit/Hyperactivity Disorder. Arch. Gen. Psychiatry 63:795. 10.1001/archpsyc.63.7.795 16818869 PMC2367150

[B38] Poldrack R. A. (2010). Interpreting developmental changes in neuroimaging signals. Hum. Brain Mapp. 31, 872–878. 10.1002/hbm.21039 20496378 PMC6870770

[B39] Posner J. Siciliano F. Wang Z. Liu J. Sonuga-Barke E. Greenhill L. (2014). A multimodal MRI study of the hippocampus in medication-naive children with ADHD: what connects ADHD and depression? Psychiatry Res. 224, 112–118. 10.1016/j.pscychresns.2014.08.006 25220159 PMC4195849

[B40] Richards J. E. Sanchez C. Phillips-Meek M. Xie W. (2016). A database of age-appropriate average MRI templates. Neuroimage 124, 1254–1259. 10.1016/j.neuroimage.2015.04.055 25941089 PMC4630162

[B41] Ross P. de Gelder B. Crabbe F. Grosbras M.-H. (2019). Emotion modulation of the body-selective areas in the developing brain. J. Dev. Cogni. Neurosci. 38:100660. 10.1016/j.dcn.2019.100660 31128318 PMC6969350

[B42] Shi F. Liu B. Zhou Y. Yu C. Jiang T. (2009). Hippocampal volume and asymmetry in mild cognitive impairment and Alzheimer's disease: Meta-analyses of MRI studies. Hippocampus 19, 1055–1064. 10.1002/hipo.20573 19309039

[B43] Sowell E. R. Jernigan T. L. (1998). Further MRI evidence of late brain maturation: limbic volume increases and changing asymmetries during childhood and adolescence. Dev. Neuropsychol. 14, 599–617. 10.1080/87565649809540731

[B44] Sung D. Park B. Kim S.-Y. Kim B.-N. Park S. Jung K.-I.. (2020). Structural alterations in large-scale brain networks and their relationship with sleep disturbances in the adolescent population. Sci. Rep. 10:3853. 10.1038/s41598-020-60692-1 32123208 PMC7051958

[B45] Takao H. Abe O. Yamasue H. Aoki S. Kasai K. Ohtomo K. (2010). Cerebral asymmetry in patients with schizophrenia: A voxel-based morphometry (VBM) and diffusion tensor imaging (DTI) study. J. Magn. Reson. Imag. 31, 221–226. 10.1002/jmri.22017 20027592

[B46] Taki Y. Hashizume H. Thyreau B. Sassa Y. Takeuchi H. Wu K.. (2012). Sleep duration during weekdays affects hippocampal gray matter volume in healthy children. NeuroImage 60, 471–475. 10.1016/j.neuroimage.2011.11.072 22197742

[B47] Tzourio-Mazoyer N. Landeau B. Papathanassiou D. Crivello F. Etard O. Delcroix N.. (2002). Automated anatomical labeling of activations in SPM using a macroscopic anatomical parcellation of the MNI MRI single-subject brain. Neuroimage 15, 273–289. 10.1006/nimg.2001.0978 11771995

[B48] Verdejo-Román J. Björnholm L. Muetzel R. L. Torres-Espínola F. J. Lieslehto J. Jaddoe V.. (2019). Maternal prepregnancy body mass index and offspring white matter microstructure: results from three birth cohorts. J. Int. J. Obes. 43, 1995–2006. 10.1038/s41366-018-0268-x 30518826

[B49] Videbech P. Ravnkilde B. (2004). Hippocampal volume and depression: a meta-analysis of MRI studies. Am. J. Psychiatry 161, 1957–1966. 10.1176/appi.ajp.161.11.1957 15514393

[B50] Wechsler D. (1974). Manual for the Wechsler Intelligence Scale for Children, Revised. New York, NY: The Psychological Corporation.

[B51] Wechsler D. (1981). Wechsler Adult Intelligence Scale-Revised Manual. New York, NY: Psychological Corporation.

[B52] Weiss Y. Booth J. R. (2017). Neural correlates of the lexicality effect in children. Brain Lang. 175, 64–70. 10.1016/j.bandl.2017.09.006 29020645 PMC5812738

[B53] Wojciulik E. Kanwisher N. (1999). The generality of parietal involvement in visual attention. Neuron 23, 747–764. 10.1016/S0896-6273(01)80033-7 10482241

[B54] Yu Q. He Z. Zubkov D. Huang S. Kurochkin I. Yang X.. (2018). Lipidome alterations in human prefrontal cortex during development, aging, and cognitive disorders. J. Mol. Psychiatry 25, 2952–2969. 10.1038/s41380-018-0200-8 30089790 PMC7577858

