# Supplementary material for: The Association Between Hippocampal Volume and Level of Attention in Children and Adolescents
Source: Front Syst Neurosci. 2021 Aug 26;15:671735. doi: 10.3389/fnsys.2021.671735 (PMC8427798; doi:10.3389/fnsys.2021.671735)
Supplement: Supplementary file 1 [file Data_Sheet_1.PDF]

Supplementary table 1. Relationships between the left (A) and right (B) hippocampal volumes and attention related variables (Partial correlation; covariates=age, sex, TIV, and IQ).

	Left Hippocampal volume			Right Hippocampal volume		
	correlation	p-values	FDR-adjusted p-values	correlation	p-values	FDR-adjusted p-value
ATAv_OE	-0.0082	0.9314	0.9884	-0.1007	0.2886	0.6756
ATAv_CE	0.096	0.3118	0.6756	0.0538	0.5716	0.9415
ATAv_RT	-0.0014	0.9884	0.9884	0.1276	0.178	0.5539
ATAv_RTv	-0.0207	0.8275	0.9791	0.0053	0.9558	0.9884
ATAa_OE	-0.2275	0.0154	0.1656	-0.2227	0.0177	0.1656
ATAa_CE	-0.0293	0.7576	0.9643	-0.008	0.933	0.9884
ATAa_RT	-0.2489	0.0079	0.1656	-0.0956	0.3137	0.6756
ATAa_RTv	-0.0845	0.3738	0.7138	0.1462	0.1223	0.4281

ATAa: Advanced Test of Attention - Auditory, ATAv: Advanced Test of Attention - Visual, OE: Omission Errors, CE: Commission Errors, RT: Mean of Response Time, RTv: Response Time variability.
